# Development and Complex Application of Methods for the Identification of Mutations in the *FAD3A* and *FAD3B* Genes Resulting in the Reduced Content of Linolenic Acid in Flax Oil

**DOI:** 10.3390/plants12010095

**Published:** 2022-12-24

**Authors:** Liubov V. Povkhova, Elena N. Pushkova, Tatiana A. Rozhmina, Alexander A. Zhuchenko, Roman I. Frykin, Roman O. Novakovskiy, Ekaterina M. Dvorianinova, Aleksey A. Gryzunov, Elena V. Borkhert, Elizaveta A. Sigova, Gleb N. Vladimirov, Anastasiya V. Snezhkina, Anna V. Kudryavtseva, George S. Krasnov, Alexey A. Dmitriev, Nataliya V. Melnikova

**Affiliations:** 1 Engelhardt Institute of Molecular Biology, Russian Academy of Sciences, 119991 Moscow, Russia; 2 Federal Research Center for Bast Fiber Crops, 172002 Torzhok, Russia; 3 All-Russian Horticultural Institute for Breeding, Agrotechnology and Nursery, 115598 Moscow, Russia; 4 Faculty of Biology, Lomonosov Moscow State University, 119234 Moscow, Russia; 5 Moscow Institute of Physics and Technology, 141701 Moscow, Russia; 6 All-Russian Scientific Research Institute of Refrigeration Industry—Branch of V.M. Gorbatov Federal Research Center for Food Systems of Russian Academy of Sciences, 127422 Moscow, Russia; 7 Skolkovo Institute of Science and Technology, 121205 Moscow, Russia

**Keywords:** flax, linseed, fatty acid composition, linolenic acid, *FAD3* genes, DNA markers, marker-assisted selection, targeted deep sequencing, HRM, CAPS

## Abstract

Flax is grown worldwide for seed and fiber production. Linseed varieties differ in their oil composition and are used in pharmaceutical, food, feed, and industrial production. The field of application primarily depends on the content of linolenic (LIN) and linoleic (LIO) fatty acids. Inactivating mutations in the *FAD3A* and *FAD3B* genes lead to a decrease in the LIN content and an increase in the LIO content. For the identification of the three most common low-LIN mutations in flax varieties (G-to-A in exon 1 of *FAD3A* substituting tryptophan with a stop codon, C-to-T in exon 5 of *FAD3A* leading to arginine to a stop codon substitution, and C-to-T in exon 2 of *FAD3B* resulting in histidine to tyrosine substitution), three approaches were proposed: (1) targeted deep sequencing, (2) high resolution melting (HRM) analysis, (3) cleaved amplified polymorphic sequences (CAPS) markers. They were tested on more than a thousand flax samples of various types and showed promising results. The proposed approaches can be used in marker-assisted selection to choose parent pairs for crosses, separate heterogeneous varieties into biotypes, and select genotypes with desired homozygous alleles of the *FAD3A* and *FAD3B* genes at the early stages of breeding for the effective development of varieties with a particular LIN and LIO content, as well as in basic studies of the molecular mechanisms of fatty acid synthesis in flax seeds to select genotypes adequate to the tasks.

## 1. Introduction

Flax (*Linum usitatissimum* L.) is one of the oldest cultivated plants, its seeds are traditionally used to produce linseed oil [1]. Flax seeds are one of the richest plant sources of healthy polyunsaturated linolenic acid and also contain a number of other valuable compounds (primarily lignans), which prevent the development of cardiovascular, oncological, and a number of other diseases [2,3,4,5,6,7,8,9,10,11]. Flax seeds are used in the production of paints, linoleum, composite materials, pharmaceuticals, food products, dietary supplements, and functional animal feed [3,6,12,13,14,15]. The main fatty acids of linseed oil are the unsaturated linolenic (LIN, ω-3), linoleic (LIO, ω-6), and oleic (OLE, ω-9) acids and saturated stearic and palmitic ones. The fatty acid composition of the oil differs significantly between varieties and determines the direction of the use of flax seeds [2,16,17,18]. Linseed oil with a high content of LIN (more than 50%) is used in the pharmaceutical and polymer industries, however, rapid oxidation limits its use in the food industry, where the seeds with a low (about 5%) and medium (30–40%) content of LIN are preferred [2,3,10,15,19,20].

For the development of flax lines with a low LIN content, mutagenesis was used. Mutant lines with a reduced content of LIN and increased content of LIO were obtained, and it was revealed that the LIN content was determined by the two genes with additive effect [21,22,23]. It is known that desaturases play a key role in the synthesis of oleic, linoleic, and linolenic acids and determine the fatty acid composition of plant oil [24]. Fatty acid desaturases 3 (FAD3) are responsible for the synthesis of LIN by the desaturation of LIO [25]. It was demonstrated that *LuFAD3A* and *LuFAD3B* are the major genes responsible for the conversion of LIO to LIN in flax, and mutations in these genes determine a low content of LIN in linseed oil [26].

Several studies aimed at finding the *FAD3A* and *FAD3B* mutations resulting in a low LIN content in linseed oil. Nonsense mutations in *FAD3A* (exon 5) and *FAD3B* (exon 1) resulted in a low content of LIN in line 593–708 [26]. Nonsense mutation in *FAD3A* (exon 2) and histidine to tyrosine substitution in *FAD3B* (exon 2) caused a low LIN content in line SP2047 [16]. Nonsense mutation in *FAD3A* (exon 5) and glycine to glutamic acid substitution in *FAD3B* (exon 5) probably prevented the conversion of LIO to LIN in the low-LIN variety TL23 [27]. Two nonsense mutations in *FAD3A* (exons 1 and 5) and a nonsense mutation and histidine to tyrosine substitution in *FAD3B* (exons 1 and 2 respectively) were revealed in flax varieties with a reduced LIN content [28]. Nonsense mutation in *FAD3A* (exon 1) and histidine to tyrosine substitution in *FAD3B* (exon 2) determined a low LIN content in the study on 40 flax lines [29]. In our previous studies on 279 flax varieties [18,30], three low-LIN mutations were identified: two nonsense mutations in *FAD3A* (exons 1 and 5) and histidine to tyrosine substitution in *FAD3B* (exon 2). Varieties with only one of the mutations had a medium content of LIN, while varieties with the mutations in both *FAD3A* and *FAD3B* had a low LIN content. Thus, inactivating mutations in the *FAD3A* and *FAD3B* genes responsible for a decrease in the content of LIN in linseed oil were identified in a significant number of studies.

Detection of low-LIN mutations in *FAD3A* and *FAD3B* can be useful for breeding flax varieties with a desired content of LIN for a particular application, especially for food purposes [20,26,28]. Marker-assisted selection is one of the most effective approaches for selecting valuable genotypes at the early stages of breeding [31]. It significantly increases the accuracy of breeding and reduces the time and material costs for creating varieties of many agricultural plants [32]. However, there is a lack of reliable test systems for the identification of low-LIN mutations in flax genotypes. Our study aimed to solve this issue.

## 2. Results

### 2.1. Selected Mutations of FAD3 Genes

The three most common mutations in the *FAD3A* and *FAD3B* genes that lead to a decrease in the LIN content and an increase in the LIO content in linseed oil were chosen for the development of test systems for their identification: G-to-A in exon 1 of *FAD3A* (CP027631.1: 16092348, GCA_000224295.2 ASM22429v2; abbreviated G-to-A/exon 1/*FAD3A*), resulting in a tryptophan to a stop codon substitution; C-to-T in exon 2 of *FAD3B* (CP027622.1: 1035655; abbreviated C-to-T/exon 2/*FAD3B*), resulting in a histidine to tyrosine substitution; and C-to-T in exon 5 of *FAD3A* (CP027631.1:16090340; abbreviated C-to-T/exon 5/*FAD3A*), resulting in an arginine to a stop codon substitution. Three approaches were proposed for the detection of these mutations in flax varieties and F_3_ hybrids: targeted deep sequencing, high-resolution melting (HRM), and cleaved amplified polymorphic sequences (CAPS) markers. The list of flax samples used in the work and approaches used for their analysis is presented in Supplementary Table S1.

### 2.2. Targeted Deep Sequencing

We used our previously proposed approach [18], which is based on the targeted deep sequencing, for the assessment of the presence/absence of the three mutations in *FAD3A* and *FAD3B* in 24 flax lines obtained by crossing flax varieties differing in oil fatty acid composition and in 19 varieties promising for the use in breeding, including those with a slightly reduced content of LIN (45–55%). G-to-A/exon 1/*FAD3A* was identified in line g.604 (about 25% of plants). C-to-T/exon 2/*FAD3B* was revealed in lines g.582 (about 65% of plants) and g.592, g.594, g.595, g.598, g.604 (about 25% of plants). C-to-T/exon 5/*FAD3A* was not found in any sample. Results of the detection of G-to-A/exon 1/*FAD3A* and C-to-T/exon 2/*FAD3B* are presented in Supplementary Table S2. Plants with both identified mutations are low-LIN and those with only one mutation are mid-LIN.

Targeted deep sequencing also enabled the screening of 94 F_3_ families from the Raciol × AGT 427/10 cross and 87 F_3_ families from the LM 98 × AGT 427/10 cross for the presence of G-to-A/exon 1/*FAD3A* and C-to-T/exon 2/*FAD3B*. Results are presented in Supplementary Table S2. F_3_ families that had homozygous mutations leading to a low or medium LIN content were chosen for further breeding flax varieties for the food industry: 44 F_3_ families for Raciol × AGT 427/10 with C-to-T/exon 2/*FAD3B*—medium LIN content; 36 F_3_ families for LM 98 × AGT 427/10 with G-to-A/exon 1/*FAD3A* or C-to-T/exon 2/*FAD3B*—medium LIN content; and 9 F_3_ families for LM 98 × AGT 427/10 with both G-to-A/exon 1/*FAD3A* and C-to-T/exon 2/*FAD3B*—low LIN content.

Moreover, we used targeted deep sequencing to detect G-to-A/exon 1/*FAD3A* and C-to-T/exon 2/*FAD3B* for 96 individual plants of line AGT 1535/07 and 96 individual plants of line AGT 987/02, for which high heterogeneity was revealed for the sites of these low-LIN mutations [18]. The results of the identification of the mutations are presented in Supplementary Table S2. The performed analysis enabled distributing the studied plants into groups with particular allelic variants of the *FAD3A* and *FAD3B* genes corresponding to a high, medium, or low LIN content and obtaining lines homogeneous by the LIN content for further use in practice and basic research.

### 2.3. HRM Analysis

**Figure 1:**
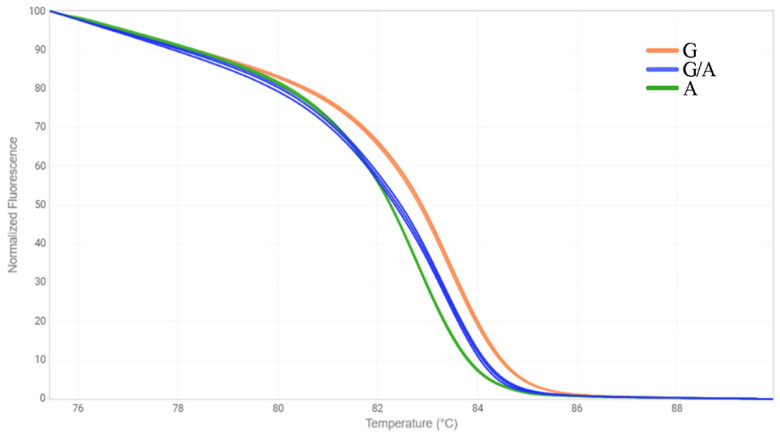
HRM analysis for the G-to-A mutation in exon 1 of *FAD3A* gene for oligonucleotide standards. G—standard without the mutation; A—standard with the mutation; G/A—standards with and without the mutation in 1:1 ratio.

**Figure 2:**
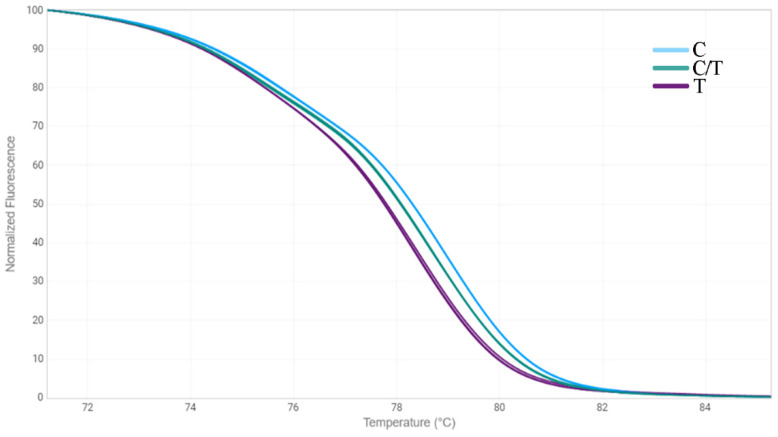
HRM analysis for the C-to-T mutation in exon 2 of *FAD3B* gene for oligonucleotide standards. C—standard without the mutation; T—standard with the mutation; C/T—standards with and without the mutation in 1:1 ratio.

**Figure 3:**
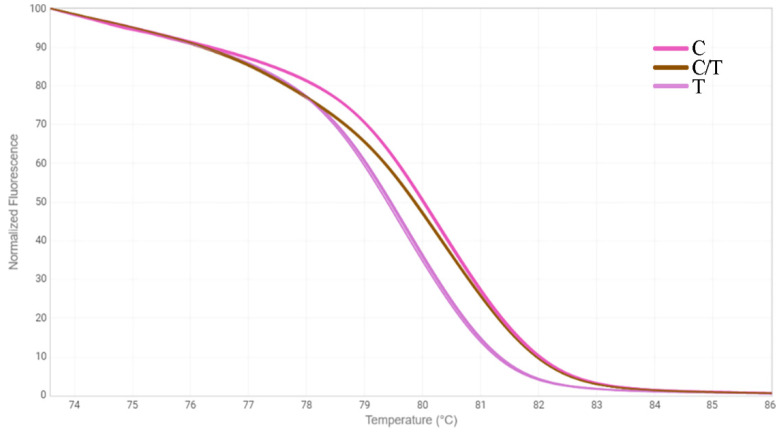
HRM analysis for the C-to-T mutation in exon 5 of *FAD3A* gene for oligonucleotide standards. C—standard without the mutation; T—standard with the mutation; C/T—standards with and without the mutation in 1:1 ratio.

**Figure 4:**
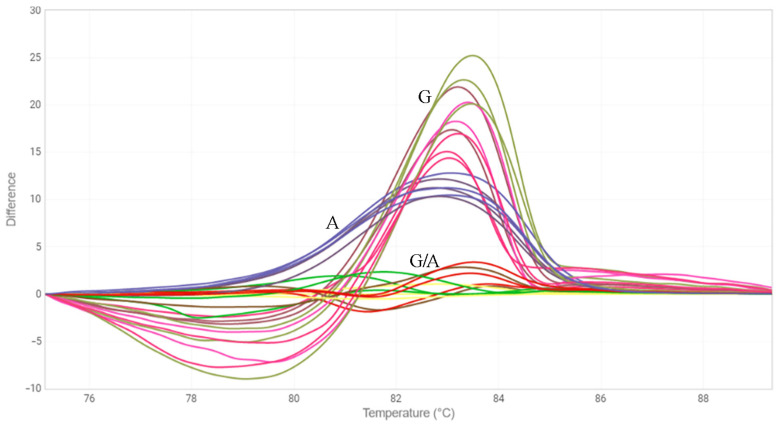
HRM analysis for the G-to-A mutation in exon 1 of *FAD3A* gene for individual plants of line AGT 1535/07. G—samples without the mutation, G/A—samples heterozygous for the mutation; A—samples with the mutation.

**Figure 5:**
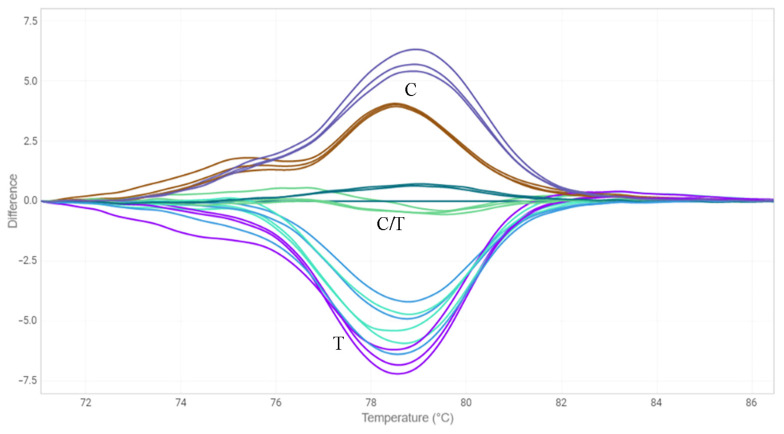
HRM analysis for the C-to-T mutation in exon 2 of *FAD3B* gene for individual plants of line AGT 981/05. C—samples without the mutation, C/T—samples heterozygous for the mutation; T—samples with the mutation.

**Figure 6:**
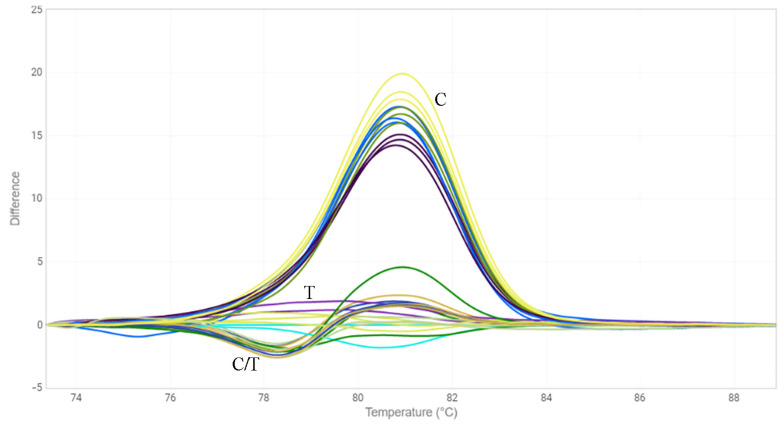
HRM analysis for the C-to-T mutation in exon 5 of *FAD3A* gene for individual plants of variety Lola. C—samples without the mutation, C/T—samples heterozygous for the mutation; T—samples with the mutation.

Another approach we chose to identify mutations in the *FAD3* genes was the HRM analysis [33]. We designed three primer pairs for the amplification of *FAD3A* and *FAD3B* regions containing G-to-A/exon 1/*FAD3A*, C-to-T/exon 2/*FAD3B*, and C-to-T/exon 5/*FAD3A* (sequences are presented in Supplementary Table S3). The primers were specific to the conserved DNA regions chosen on the basis of our previous studies on polymorphisms of the *SAD* and *FAD* genes in 279 flax varieties [18,30]. Six oligonucleotide standards were also developed, they corresponded to the variants of *FAD3A* and *FAD3B* with and without the analyzed mutations (Supplementary Table S3). PCR and HRM conditions were optimized. Validation of the developed test systems was carried out using oligonucleotide standards (Figure 1, Figure 2 and Figure 3) and individual plants of heterogeneous flax varieties, which were already subjected to the targeted deep sequencing analysis (AGT 1535/07 and AGT 987/02, 92 plants for each) (Figure 4, Figure 5 and Figure 6). Homozygous samples with and without the mutations and heterozygous samples were clearly distinguished from each other by the temperature shift and shape change of the DNA melting curves. The results of the mutation identification in the *FAD3A* and *FAD3B* genes obtained using deep sequencing and HRM analysis were in complete agreement. Thus, genotypes with the *FAD3A* and *FAD3B* allelic variants corresponding to a high, medium, or low LIN content were chosen from varieties AGT 1535/07 and AGT 987/02. HRM was also used for the selection of proper genotypes for further transcriptomic studies of heterogeneous varieties Lola, AGT 981/05, and AGT 1535/07 (40 individual plants for each variety). It enabled the identification and exclusion of non-typical plants from further research: 9 for Lola, 4 for AGT 981/05, and 15 for AGT 1535/07.

### 2.4. CAPS Markers

**Figure 7:**
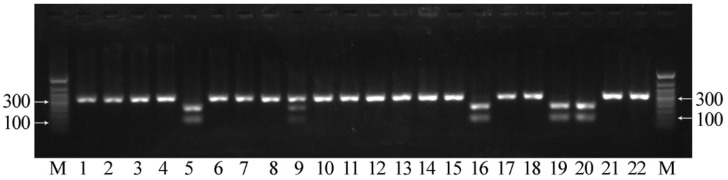
Results of CAPS analysis for the G-to-A mutation in exon 1 of *FAD3A* for individual plants of line AGT 1535/07. If the mutation is absent, two fragments with the lengths of 195 and 107 bp were visualized (lines 5, 16, 19, 20), while if the mutation is present, one fragment with a length of 302 bp was visualized (lines 1–4, 6–8, 10–15, 17, 18, 21, 22). In the case of heterozygosity for the studied mutation, three DNA fragments were visualized, corresponding to the initial length of the amplicon and two of its parts after restriction (line 9). M—50 bp ladder.

**Figure 8:**
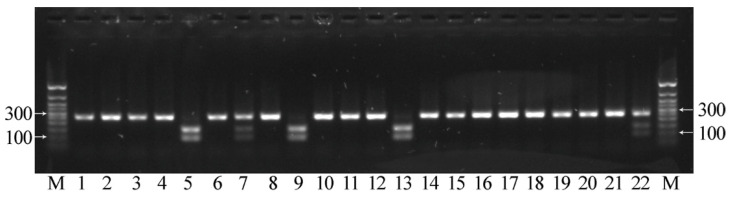
Results of CAPS analysis for the C-to-T mutation in exon 2 of *FAD3B* for individual plants of line AGT 981/05. If the mutation is absent, two fragments with the lengths of 157 and 103 bp were visualized (lines 5, 9, 13), while if the mutation is present, one fragment with a length of 260 bp was visualized (lines 1–4, 6, 8, 10–12, 14–21). In the case of heterozygosity for the studied mutation, three DNA fragments were visualized, corresponding to the initial length of the amplicon and two of its parts after restriction (lines 7, 22). M—50 bp ladder.

**Figure 9:**
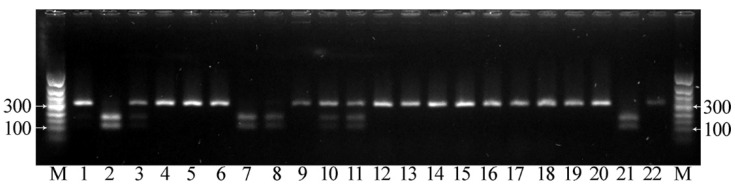
Results of CAPS analysis for the C-to-T mutation in exon 5 of *FAD3A* for individual plants of variety Lola. If the mutation is absent, two fragments with the lengths of 163 and 109 bp were visualized (lines 2, 7, 8, 21), while if the mutation is present, one fragment with a length of 272 bp was visualized (1, 4–6, 9, 12–20, 22). In the case of heterozygosity for the studied mutation, three DNA fragments were visualized, corresponding to the initial length of the amplicon and two of its parts after restriction (3, 10, 11). M—50 bp ladder.

We also developed an approach for the identification of the three *FAD3A* and *FAD3B* mutations based on CAPS markers. This analysis includes the amplification of the target gene region with subsequent cleavage with restriction enzymes and evaluation of the DNA fragment lengths by gel electrophoresis [34]. We designed primers for the amplification of *FAD3A* and *FAD3B* regions containing the studied mutations (sequences are presented in Supplementary Table S3). As in the HRM analysis, primers were specific to the conserved regions of flax DNA. After PCR, in the absence of G-to-A/exon 1/*FAD3A* and C-to-T/exon 2/*FAD3B* (high LIN content), the resulting amplicons contained the HaeIII restriction site (GG∨CC/CC^GG) at the sites of these mutations. Amplicons after the restriction were separated by gel electrophoresis, and two DNA fragments were visualized in each case. However, in the presence of the studied mutations, the HaeIII restriction site in the amplicons was absent, and only one fragment was observed in each case (Figure 7 and Figure 8). In the absence of C-to-T/exon 5/*FAD3A*, the resulting amplicon contained the BstMBI restriction site (∨GATC/CTAG^), and two DNA fragments were visualized. However, in the presence of this mutation, the BstMBI restriction site was absent in the amplicon, and only one fragment was observed (Figure 9). In the case of heterozygosity of the studied mutations, three DNA fragments were visualized, corresponding to the initial length of the amplicon and two of its parts after restriction (Figure 7, Figure 8 and Figure 9).

CAPS markers were used for the identification of G-to-A/exon 1/*FAD3A* and C-to-T/exon 2/*FAD3B* in 185 individual plants of line AGT 1535/07 and 162 individual plants of line AGT 987/02. In these lines, high heterogeneity in the sites carrying low-LIN mutations was previously revealed [18]. From the examined sample set, 96 plants of AGT 1535/07 and 96 plants of AGT 987/02 were already studied for the presence of mutations in the *FAD3A* and *FAD3B* genes by the targeted deep sequencing, and the results of the two approaches were concordant. The application of CAPS markers made it possible to select plants of lines AGT 1535/07 and AGT 987/02 with homozygous allelic variants of the *FAD3A* and *FAD3B* genes corresponding to a high, medium, or low LIN content. The obtained groups of genotypes provide a perspective for the further development of flax varieties for specialized use in industry, food, and pharmaceuticals.

The developed CAPS markers were also used for the assessment of the presence of G-to-A/exon 1/*FAD3A*, C-to-T/exon 2/*FAD3B*, and C-to-T/exon 5/*FAD3A* in 600 samples of flax varieties Lola, AGT 981/05, and AGT 1535/07, which we used for transcriptomic studies of the mechanisms responsible for the linseed oil composition. It was extremely important as non-typical genotypes could distort the results of the analysis and lead to incorrect conclusions. Typical plants of Lola should have one of the three studied mutations—C-to-T/exon 5/*FAD3A*—while plants of AGT 981/05 and AGT 1535/07 should have two mutations—G-to-A/exon 1/*FAD3A* and C-to-T/exon 2/*FAD3B*. As a result, 42 of 200 Lola samples, 20 of 200 AGT 981/05 samples, and 81 of 200 AGT 1535/07 samples were excluded from further analysis, as they did not have the necessary mutations, or heterozygosity was revealed. Thus, a representative set of flax genotypes was formed for the assessment of gene expression in plants with a reduced LIN content, as required by the planned research.

## 3. Discussion

The content of unsaturated fatty acids in oil is one of the most important characteristics of flax. In the classical breeding of linseed, estimation of the fatty acid composition of the oil is usually carried out by the chromatographic method [35]. However, this method has several disadvantages: it often requires a significant number of seeds for the analysis; the use of a pool of heterogeneous seeds leads to an incorrect determination of oil fatty acid composition for a variety; the method does not allow differentiation between homozygous and heterozygous genotypes. Therefore, the assessment of fatty acid composition is usually performed only at the later stages of flax breeding, which makes the creation of varieties with the desired content of LIN a complex and lengthy process. The detection of *FAD3A* and *FAD3B* mutations that lead to a decrease in the LIN content can solve the indicated problems. It will enable the analysis of individual plants using only a few leaves for DNA isolation, making it possible to perform other studies of this genotype and obtain its seeds. It will also allow distinguishing between the mutations in heterozygous and homozygous states. Often, the use of data on the *FAD3* mutations is the only reliable way to reasonably select material for flax breeding or research. For example, the medium content of LIN in linseed oil can be determined by any of the several mutations in the *FAD3A* or *FAD3B* genes. By phenotype, it is impossible to resolve which mutation is present in a variety. Choosing mid-LIN varieties for crossing to create low-LIN varieties, it is necessary that one parent carries the mutation in *FAD3A* and the other in *FAD3B*. This can be discovered from the sequences of these genes but not from the content of LIN in the oil. In the case of basic research, data on which mutations determine a reduced LIN content are necessary to create a representative set of varieties. This also cannot be carried out based on the analysis of the oil fatty acid composition.

For the identification of *FAD3* mutations that result in a low LIN content in linseed oil, only CAPS markers were previously used [26,29,36]. Perhaps, the CAPS analysis was the only method because it is easy-to-use and can be applied in a wide range of laboratories, including those where high-throughput sequencers and real-time PCR systems are unavailable [37,38]. CAPS markers were designed for the detection of nonsense mutations in *FAD3A* (exon 5) and *FAD3B* (exon 1) [26], nonsense mutation in *FAD3A* (exon 5) [39], nonsense mutations in *FAD3A* (exon 1) and histidine to tyrosine substitution in *FAD3B* (exon 2) [29]. However, in the study [39], authors failed to identify low-LIN varieties using the developed CAPS markers. Probably, the studied varieties had other low-LIN mutations. In the present work, we developed CAPS markers for three low-LIN mutations of flax, which we identified earlier in the valuable-for-breeding flax varieties: G-to-A/exon 1/*FAD3A*, C-to-T/exon 2/*FAD3B*, and C-to-T/exon 5/*FAD3A* [18,30]. In developing the test system, we took into account the data on polymorphisms in about 300 flax varieties [18,30]. This enabled us to design primers that were specific to the conserved regions of flax DNA. Therefore, they are suitable for a wide range of flax genotypes. In addition, we designed primers in such a way that the amplified fragments were relatively short in length—250–300 bp. The amplification of longer fragments, as suggested in the works [26,29,36], can increase the requirements for DNA quality and PCR conditions. Thus, the developed CAPS markers can be applied in the analysis of diverse flax genotypes and allows the use of even low-quality DNA. Moreover, we developed two throughput approaches for the identification of the three low-LIN mutations in flax. They are based on the HRM analysis and targeted deep sequencing.

HRM analysis is widely used to identify DNA polymorphisms and genotype samples [40,41,42,43]. HRM does not require the use of expensive fluorophore-labeled SNP-specific probes in contrast to TaqMan assays [44,45]. The cost of the HRM analysis is usually slightly lower than that of another widely used fluorescence-based method—kompetitive allele specific PCR (KASP) [46,47,48,49,50]. Therefore, the use of HRM for plant genotyping could be a reasonable approach [51]. In our work, we developed the HRM-based test system for identifying the three mutations in the *FAD3A* and *FAD3B* genes that determine the content of LIN. HRM has a lower risk of contamination compared to CAPS since the analysis is carried out on real-time PCR platforms and there is no need to open tubes with amplicons. HRM is also faster than CAPS, it allows the assessment of a large sample set in a short time (only few hours are required).

High-throughput sequencing is a powerful tool for studying genetic diversity, developing molecular markers, and introducing new methods to plant breeding [52,53,54,55,56,57]. This technology is actively used in molecular genetic studies of flax using various approaches [58,59,60,61,62,63,64,65,66,67,68,69,70,71,72,73,74,75,76,77,78,79,80,81,82,83,84,85,86,87,88,89,90,91,92]. Along with microarrays [93,94,95], this method is the most throughput, but the second-generation sequencing (Illumina, BGI, and other platforms [96]) is much more precise than microarrays. Targeted deep sequencing is based on the enrichment of the regions of interest (PCR with target-specific primers in our case) and sequencing of a large number of samples in one run. This approach significantly reduces the cost of sequencing per genotype compared to the whole genome analysis and allows the sequencing of dozens and hundreds of genome regions in hundreds and thousands of samples. It also overcomes the difficulties of Sanger sequencing associated with polyploidy of plant genomes and a high degree of duplication of their genes [18,97,98,99,100,101]. Therefore, the third approach, developed by us, for the identification of the three low-LIN mutations in the *FAD3A* and *FAD3B* genes in flax genotypes was based on targeted deep sequencing. Compared to the HRM analysis and CAPS markers, the main advantage of targeted deep sequencing for the identification of the *FAD3* mutations was its high accuracy and the ability to assess sample heterogeneity. For this method, a sample can be either an individual plant or a pool of dozens or hundreds of plants of the same variety.

In our work, the costs of HRM-based and CAPS-based approaches were comparable, but the targeted deep sequencing was several times more expensive per sample. The advantage of HRM and CAPS is the capacity to analyze only a few samples at a time. Targeted deep sequencing has an unmatched accuracy, it is designed for large sample sets and allows assessing many polymorphisms at once. In the case of screening studies of several thousand samples in one run, the cost of the analysis per sample performed using targeted deep sequencing will be close to those performed using HRM and CAPS.

The complex application of the three proposed approaches for the identification of the *FAD3* mutations makes it possible to increase the efficiency of flax breeding and the accuracy of scientific research on this species. It is advisable to start the analysis of a collection of flax varieties of interest with targeted deep sequencing. For each variety, a DNA pool of several dozens or hundreds of plants should be analyzed to determine the presence of the *FAD3* gene mutations and assess the varietal heterogeneity (the mutations may be present only in a subset of plants of variety). On the basis of the obtained data, it is possible to select the most suitable genotypes for further work. These can be promising parental pairs for crossing, heterogeneous varieties whose purity needs to be improved, and genotypes valuable for basic research. Moreover, targeted deep sequencing could be effective to develop DNA certificates of varieties and control the varietal purity when maintaining collections and seed production of flax.

The use of HRM and CAPS markers is advisable for choosing individual plants in the marker-assisted selection of linseed, as well as plants with a desired genotype for basic research. The HRM and CAPS approaches enable the identification of the three most common low-LIN mutations in the *FAD3* genes already in F_2_ hybrids (and hybrids of later generations) and the prediction of the LIN content in seeds of these plants. Therefore, even at the early stages of breeding, it is possible to select genotypes with desirable allelic variants of the *FAD3A* and *FAD3B* genes in the homozygous state and continue working only with these genotypes. In addition, the use of HRM and CAPS markers is effective for increasing the purity of varieties for which heterogeneity in the *FAD3* gene mutations was identified.

Thus, based on the targeted deep sequencing, HRM analysis, and CAPS markers, we developed reliable approaches for the identification of the three most common mutations in the *FAD3A* and *FAD3B* genes that determine a reduced content of LIN in linseed oil: G-to-A/exon 1/*FAD3A*, C-to-T/exon 2/*FAD3B*, and C-to-T/exon 5/*FAD3A*. The choice of one of the methods or their combinations depends on the task being solved, the material being assessed, and the availability of the equipment. Using different combinations of the developed approaches, we successfully analyzed more than a thousand of flax samples and selected plants with the desired stably inherited LIN and LIO content for breeding flax varieties for specialized purposes, as well as studying molecular mechanisms related to the fatty acid synthesis.

## 4. Materials and Methods

### 4.1. Plant Material

The following flax samples from the collection of the Institute for Flax (Torzhok, Russia) were used in the work (the list of samples and methods is presented in Supplementary Table S1):

1. A total of 24 lines (g.579, g.580, g.582, g.586, g.587, g.588, g.591, g.592, g.594, g.595, g.597, g.598, g.602, g.603, g.604, g.605, g.610, g.614, g.615, g.616, g.618, g.619, g.620, g.621) obtained from crossing flax varieties differing in the fatty acid composition of the oil and 19 varieties promising for use in breeding, including those with a slightly reduced LIN content (45–55%): Hi de France, L.8709-5-10, N 3860, B-15, N 3895, K-74, Moolzyzan, Cki 10, NP RR, Soletsky kr, S Yanyshina A.A., T Yanyshina A.A., N 3810, N 3841, Stormont Motley, N 3809, Istok, Honkie 49, Focus. For each sample, material was obtained from pools of 50 or more plants;
2. Families of F_3_ hybrids of flax obtained from crosses Raciol × AGT 427/10 (94 F_3_ families) and LM 98 × AGT 427/10 (87 F_3_ families) with a contrast ratio of LIN and LIO in linseed oil and different allelic variants of the *FAD3A* and *FAD3B* genes. For each F_3_ family, material was obtained from a pool of at least 20 plants;
3. Varieties AGT 1535/07 (185 individual plants) and AGT 987/02 (162 individual plants) for which high heterogeneity was revealed for sites of low-LIN mutations [18]. Material was obtained from each individual plant;
4. Varieties Lola, AGT 981/05, and AGT 1535/07 (200 samples for each variety), which we used for transcriptomic studies of the mechanisms of linseed oil synthesis and for which some heterogeneity was revealed for sites of the low-LIN mutations [18]. Material was obtained from each individual plant.

### 4.2. DNA Isolation

Leaves were used for DNA extraction. They were ground using a MagNA Lyser (Roche, Basel, Switzerland) or TissueLyser II (Qiagen, Chatsworth, CA, USA) homogenizers. Thereafter, the CTAB method (in case of pools of several plants) or DNA-Extran-3 kit (Syntol, Moscow, Russia) (in case of individual plants) were used for DNA extraction. DNA quality was assessed by electrophoresis in 1% agarose gel. DNA concentration was determined with a Qubit fluorometer (Thermo Fisher Scientific, Waltham, MA, USA) or a NanoDrop spectrophotometer (Thermo Fisher Scientific).

### 4.3. Targeted Deep Sequencing

To prepare DNA libraries for the targeted deep sequencing, an approach based on two sequential PCR was used: the first PCR was to amplify selected gene regions and add universal adapter sequences to them, and the second PCR was to add sequences required for high-throughput sequencing and barcodes for sample identification [18]. For amplification of sites containing the G-to-A mutation in exon 1 of *FAD3A* (CP027631.1: 16092348, GCA_000224295.2 ASM22429v2; tryptophan to a stop codon substitution), C-to-T mutation in exon 2 of *FAD3B* (CP027622.1: 1035655; histidine to tyrosine substitution), and C-to-T mutation in exon 5 of *FAD3A* (CP027631.1: 16090340; arginine to a stop codon substitution), we used primers, as proposed by us earlier (sequences are presented in Supplementary Table S3), and a protocol for DNA library preparation for targeted deep sequencing [18]. One primer pair enabled simultaneous amplification of the *FAD3A* region carrying the G-to-A mutation and *FAD3B* region carrying the C-to-T mutation, while another primer pair amplified the *FAD3A* region with the C-to-T mutation. The quality and concentration of the obtained DNA libraries were evaluated on a 2100 Bioanalyzer (Agilent Technologies, Santa Clara, CA, USA) and Qubit fluorometer (Thermo Fisher Scientific). The resulting pools of DNA libraries were sequenced on MiSeq (Illumina, San Diego, CA, USA) using the MiSeq Reagent Kit v3 (600 cycles). For deep sequencing data processing, reads were trimmed and filtered using Trimmomatic [102], mapped to the reference genome of flax variety Bethune (GCA_000224295.2 ASM22429v2) using BWA-MEM [103]. Mapped reads were grouped using Picard, and polymorphisms were searched in the regions of interest using the FreeBayes tool [104].

### 4.4. HRM Analysis

For the HRM analysis of *FAD3A* and *FAD3B* gene regions containing the sites of the three studied mutations determining the content of LIN and LIO (G-to-A in exon 1 of *FAD3A* (CP027631.1: 16092348, GCA_000224295.2 ASM22429v2), C-to-T in exon 2 of *FAD3B* (CP027622.1: 1035655), and C-to-T in exon 5 of *FAD3A* (CP027631.1: 16090340)), primers (Supplementary Table S3) were designed for the conserved regions taking into account the data on polymorphisms obtained by us earlier for 279 flax varieties [18,30]. Reaction mix was prepared in a volume of 20 μL for each sample and standard as follows: 1× Taq Turbo buffer (Evrogen, Moscow, Russia), 250 nM of dNTPs (Evrogen), 350 nM of each primer, 1× EvaGreen dye (Biotium, Fremont, CA, USA), 1× LowROX dye (Evrogen), 2 units of HS Taq DNA polymerase (Evrogen). DNA concentration was about 5 ng/µL for samples and 5 fg/µL for standards. Synthesized oligonucleotides were used as standards (with and without each of the three analyzed mutations, Supplementary Table S3). The following amplification program was used: 95 °C—10 min; 50 cycles of 95 °C—15 s, 60 °C—1 min. Each reaction was performed in triplicate. Then, the HRM analysis was carried out: 65–90 °C, step 0.1 °C. Data processing was performed using the QuantStudio 5 (Thermo Fisher Scientific) software. For each low-LIN mutation, the obtained melting curves for the studied flax samples were compared with the melting curves for two standards (with and without the mutation) and a 1:1 mixture of the two standards. The similarity between the melting curves of the examined sample and the standard carrying the mutation indicated the presence of the mutation in the studied genotype in the homozygous state. The similarity between the melting curves of the examined sample and the standard without the mutation indicated the absence of the mutation in the studied genotype. The similarity between the melting curves of the examined sample and the 1:1 mixture of two standards indicated the presence of the mutation in the studied genotype in the heterozygous state.

### 4.5. CAPS Markers

To conduct the CAPS analysis of *FAD3A* and *FAD3B* gene regions containing the sites of three studied mutations that determine the content of LIN and LIO, primers (Supplementary Table S3) were designed for the conserved regions of these genes, taking into account the data on polymorphisms obtained by us earlier for 279 flax varieties [18,30]. For amplification in a volume of 15 µL, the following reaction mixture was used: 1× Taq Turbo buffer (Evrogen), 250 nM of dNTPs (Evrogen), 350 nM of each primer, 1 unit of HS Taq DNA polymerase (Eurogen), 20 ng of DNA. The following amplification program was used: 95 °C—3 min; 40 cycles of 95 °C—30 s, 60 °C—30 s, 72 °C—60 s; 72 °C—3 min. Then, restriction was performed for 4 μL of each PCR product. In the case of the G-to-A mutation in exon 1 of *FAD3A* (CP027631.1: 16092348, GCA_000224295.2 ASM22429v2) and C-to-T mutation in exon 2 of *FAD3B* (CP027622.1: 1035655), incubation with 10 units of HaeIII (SibEnzyme, Novosibirsk, Russia, restriction site GG∨CC/CC^GG) was performed at 37 °C for 4 h followed by inactivation of the restriction enzyme by heating to 80 °C for 20 min. In the case of the C-to-T mutation in exon 5 of *FAD3A* (CP027631.1: 16090340), incubation with 10 units of BstMBI (SibEnzyme, restriction site ∨GATC/CTAG^) at 65 °C for 4 h followed by inactivation of the restriction enzyme by heating to 80 °C for 20 min was performed. Restriction products were visualized by 2% agarose gel electrophoresis. In the absence of the G-to-A mutation in exon 1 of *FAD3A*, the lengths of the fragments were 195 and 107 bp, while in the presence of the mutation, the fragment length was 302 bp. In the absence of the C-to-T mutation in exon 2 of *FAD3B*, the lengths of the fragments were 157 and 103 bp, while in the presence of the mutation, the fragment length was 260 bp. In the absence of the C-to-T mutation in exon 5 of *FAD3A*, the lengths of the fragments were 163 and 109 bp, and in the presence of the mutation, the fragment length was 272 bp.

## Data Availability

The raw sequencing data have been deposited in the NCBI Sequence Read Archive (SRA) under the BioProject accession number PRJNA625974.

## Supplementary Materials

The following supporting information can be downloaded at: https://www.mdpi.com/article/10.3390/plants12010095/s1, Table S1: Flax plant material from the collection of the Institute for Flax that was used for the identification of the G to A mutation in exon 1 of *FAD3A*, C to T mutation in exon 2 of *FAD3B*, and C to T mutation in exon 5 of *FAD3A* using targeted deep sequencing, high resolution melting (HRM) analysis, and cleaved amplified polymorphic sequences (CAPS) markers. Table S2: Results of the identification of the G to A mutation in exon 1 of *FAD3A* and C to T mutation in exon 2 of *FAD3B* in flax samples using targeted deep sequencing. Table S3: Sequences of primers and oligonucleotide standards for targeted deep sequencing, HRM analysis, and CAPS markers developed for the identification of the G to A mutation in exon 1 of *FAD3A*, C to T mutation in exon 2 of *FAD3B*, and C to T mutation in exon 5 of *FAD3A*.
