# Supplementary material for: Development and Complex Application of Methods for the Identification of Mutations in the FAD3A and FAD3B Genes Resulting in the Reduced Content of Linolenic Acid in Flax Oil
Source: Plants (Basel). 2022 Dec 24;12(1):95. doi: 10.3390/plants12010095 (PMC9824437; doi:10.3390/plants12010095)
Supplement: Supplementary file 1 [file plants-12-00095-s001.zip › Supplementary Table S3.pdf]

Supplementary Table S3. Sequences of primers and oligonucleotide standards for targeted deep sequencing, HRM analysis, and CAPS markers developed for the identification of the G to A mutation in exon 1 of *FAD3A*, C to T mutation in exon 2 of *FAD3B*, and C to T mutation in exon 5 of *FAD3A*.

Mutation	Primer name	Sequence
**Targeted deep sequencing**		
G to A in exon 1 of *FAD3A* and C to T in exon 2 of *FAD3B*	DeepSeq_FAD3_4_F	TCGTCGGCAGCGTCAGATGTGTATAAGAGACAGTGGGTGAAGAACCCCTGGA
	DeepSeq_FAD3_4_R	GTCTCGTGGGCTCGGAGATGTGTATAAGAGACAGTCTTTCTCCACATTGCCGTGA
C to T in exon 5 of *FAD3A*4	DeepSeq_FAD3_9_F	TCGTCGGCAGCGTCAGATGTGTATAAGAGACAGCCACGGGTACGAGCAGAAG
	DeepSeq_FAD3_9_A_R	GTCTCGTGGGCTCGGAGATGTGTATAAGAGACAGAGGSTGATCAYGTGGGTGAA
**High resolution melting (HRM) analysis**		
G to A in exon 1 of *FAD3A*	HRM_FAD3A_E1_F	AAGCTGGACAGCTGGACTG
	HRM_FAD3A_E1_R	CAGAACATGGTTCCTTGAGCAAT
	HRM_FAD3A_E1_ref	AAGCTGGACAGCTGGACTGTCTGGCCTCTCTACTGGATTGCTCAAGGAACCATGTTCTG
	HRM_FAD3A_E1_alt	AAGCTGGACAGCTGGACTGTCTGACCTCTCTACTGGATTGCTCAAGGAACCATGTTCTG
C to T in exon 5 of *FAD3A*	HRM_FAD3A_E5_F	GAGGGCTGACGACCGTC
	HRM_FAD3A_E5_R	CATTTGAGGGAAGAGATGGTGAATA
	HRM_FAD3A_E5_ref	GAGGGCTGACGACCGTCGATCGAGATTACGGGGTCATCAACACCATCCACCATGACATTGGCACCCATGTTATTCACCATCTCTTCCCTCAAATG
	HRM_FAD3A_E5_alt	GAGGGCTGACGACCGTCGATTGAGATTACGGGGTCATCAACACCATCCACCATGACATTGGCACCCATGTTATTCACCATCTCTTCCCTCAAATG
C to T in exon 2 of *FAD3B*	HRM_FAD3B_E2_F	ATCTGGGTTGTTGCATGC
	HRM_FAD3B_E2_R	TGTCTGAGAAGCTCCCAT
	HRM_FAD3B_E2_ref	ATCTGGGCTGTTGCATGCAGTGGCCATGGGAGCTTCTCAGACA
	HRM_FAD3B_E2_alt	ATCTGGGCTGTTGCATGCAGTGGCTATGGGAGCTTCTCAGACA
**Cleaved amplified polymorphic sequences (CAPS) markers**		
G to A in exon 1 of *FAD3A*	CAPS_FAD3A_exon 1_F	TCAATGGGGCGAAGAAGCTA
	CAPS_FAD3A_exon 1_R	AGAAAAGAAAACCCACATTACCAGA
C to T in exon 5 of *FAD3A*	CAPS_FAD3A_exon 5_F	GGCTTCCTTCCGGTCTTCAA
	CAPS_FAD3A_exon 5_R	GACCTCCTTACCGCTTCGAC
C to T in exon 2 of *FAD3B*	CAPS_FAD3B_exon 2_F	TGTTCTTGGACATGATTGGTAAACT
	CAPS_FAD3B_exon 2_R	AAATTAGGGTTTGGATTCTTCAGC

Note: The G to A mutation in exon 1 of *FAD3A* gene is located on CP027631.1: 16092348 (according to the assembly of cultivar CDC Bethune – GCA_000224295.2 ASM22429v2) and results in tryptophan with a stop codon substitution; the C to T mutation in exon 5 of *FAD3A* gene is located on CP027631.1: 16090340 and results in arginine with a stop codon substitution; the C to T mutation in exon 2 of *FAD3B* gene is located on CP027622.1: 1035655 and results in histidine with tyrosine substitution.
